# Genetic susceptibility and gene–environment interactions in gastric cancer among ethnic populations of Northeast India

**DOI:** 10.1038/s41598-026-50133-w

**Published:** 2026-05-06

**Authors:** Kangjam Rekha Devi, Debdutta Mukherjee, Sanjib Phukan, Mandakini Gogoi, Gautam Majumdar, V. Khamo, Kanwar Narain

**Affiliations:** 1 Scientist-G, ICMR-RMRC, Dibrugarh, Assam India; 2 ICMR-RMRC, Dibrugarh, Assam India; 3 Project Scientist -II, ICMR-RMRC, Dibrugarh, Assam India; 4 Former medical superintendent of the Regional Cancer Centre, Agartala, India; 5 Naga Hospital Authority, Kohima, Nagaland India; 6 Former Director, ICMR-RMRC, Dibrugarh, Assam India

**Keywords:** Gastric cancer, Germline mutations, Genetic polymorphisms, Gene-environment interaction, Wnt signalling pathway, TP53, Northeast India, Betel nut chewing, Cancer, Diseases, Gastroenterology, Genetics, Oncology, Risk factors

## Abstract

Northeast India has a high burden of gastric cancer, but the factors driving this elevated risk remain poorly understood. This study examined genetic polymorphisms, germline mutations, and lifestyle factors contributing to gastric cancer risk in populations from Nagaland and Tripura. We conducted a case–control study of 190 gastric cancer patients recruited at the hospitals and 317 healthy community controls, integrating PCR-based genotyping of *GSTM1*,* GSTT1*,* CYP2E1*,* TLR2*,* TLR4*,* TNF-α*, and *IL-10* with detailed assessment of lifestyle, diet, and *Helicobacter pylori* infection, alongside ARMS^®^-based qBiomarker™ mutation arrays for sensitive detection of germline cancer-associated variants. The GSTM1 null genotype was associated with a significantly increased risk of gastric cancer (adjusted OR 2.26, 95% CI 1.49–3.43; *p* < 0.001). Among ever tobacco chewers, individuals carrying the *GSTM1*-null genotype had a markedly higher risk than tobacco chewers carrying the *GSTM1* non-null genotype (adjusted OR 12.63, 95% CI 3.04–52.42; *p* < 0.001). Regular consumption of pumpkin, tomatoes, cabbage, and seasonal fruits reduced gastric cancer risk by 40–70%. Germline mutations were frequent in *TP53* (81.25%), *CTNNB1* (71.25%), *APC* (53.75%), and *CDKN2A* (31.25%), suggesting possible inherited susceptibility and founder mutations in these populations. This study highlights strong gene–environment interactions, frequent germline mutations, and the protective role of diet in gastric cancer risk. The findings emphasize the need for population-specific genetic screening, counselling, and preventive strategies tailored to Northeast India’s unique populations.

**Supplementary Information:**

The online version contains supplementary material available at 10.1038/s41598-026-50133-w.

## Introduction

Cancer continues to constitute a major public-health burden in India, with an estimated 1.4–1.5 million new diagnoses recorded annually^1^. Recent data from the Population-Based Cancer Registries (PBCR 2020) revealed a striking geographical heterogeneity in India. A very high incidence of gastric cancer is reported from the Northeastern states of India^2^. The incidence (age-adjusted) of gastric cancer reaches 17.91 per 100,000 in males and 11.84 per 100,000 in females in Nagaland, whereas Tripura records lower, yet still substantial, rates of 5.0 per 100,000 in males and 2.15 per 100,000 in females. The high incidence of gastric cancer in Northeastern states may be attributed to an intricate interplay of hereditary predispositions, lifestyle practices—including tobacco, betel-nut use, and distinctive dietary habits—and unique environmental exposures such as pesticide burden in the North-East^3–7^.

The human xenobiotic-metabolising system, comprising phase I and phase II enzymes, protects against carcinogenic insults encountered through inhalation, ingestion, or dermal contact^8–11^. Phase I cytochrome P450 (CYP) enzymes bio-activate pro-carcinogens, whereas phase II conjugating enzymes—notably the glutathione-S-transferases (GSTs), N-acetyltransferases, and sulfotransferases—detoxify reactive intermediates and facilitate excretion^12–14^. Polymorphic variation in genes encoding these enzymes (e.g., *GSTM1*, *GSTT1*, *CYP2E1*) modulates enzymatic efficiency and thereby influences individual cancer susceptibility^3,4,15,16^.

Beyond detoxification, host immunity plays a crucial role in shaping gastric carcinogenesis. Polymorphisms in Toll-like receptor genes (*TLR2*, *TLR4*) have been reported to increase cancer risk^6,17,18^. Likewise, variants in pro- and anti-inflammatory cytokine genes—*TNF-α* and *IL-10*—have been associated with heightened risk of cancer, including gastric cancer susceptibility^19–21^.

At the somatic and germline level, driver alterations in oncogenes and tumour-suppressor genes orchestrate malignant transformation^22,23^. Despite mounting evidence linking genetic polymorphisms, immune dysregulation, and environmental exposures to gastric cancer, comprehensive studies in North-Eastern ethnic groups remain scarce. The present investigation, therefore, seeks to bridge this gap by assessing polymorphisms in *GSTM1*, *GSTT1*, and *CYP2E1* and their interaction with tobacco use and betel-nut chewing in Nagaland and Tripura. Evaluating *TLR2* (−196/−174 del) and *TLR4* (Asp299Gly, Thr399Ile) polymorphisms in relation to gastric cancer risk. Determining the influence of *IL-10* and *TNF-α* cytokine variants on disease susceptibility. Measuring sero-prevalence of *Helicobacter pylori* among cases and controls. Profiling mutations in key gastric-cancer driver genes through targeted PCR arrays. A clearer understanding of these genetic and environmental determinants will facilitate the development of population-tailored screening panels, personalised risk stratification, and preventive strategies for gastric cancer in India’s North-East.

## Methods

### Study design and population

**Fig. 1:**
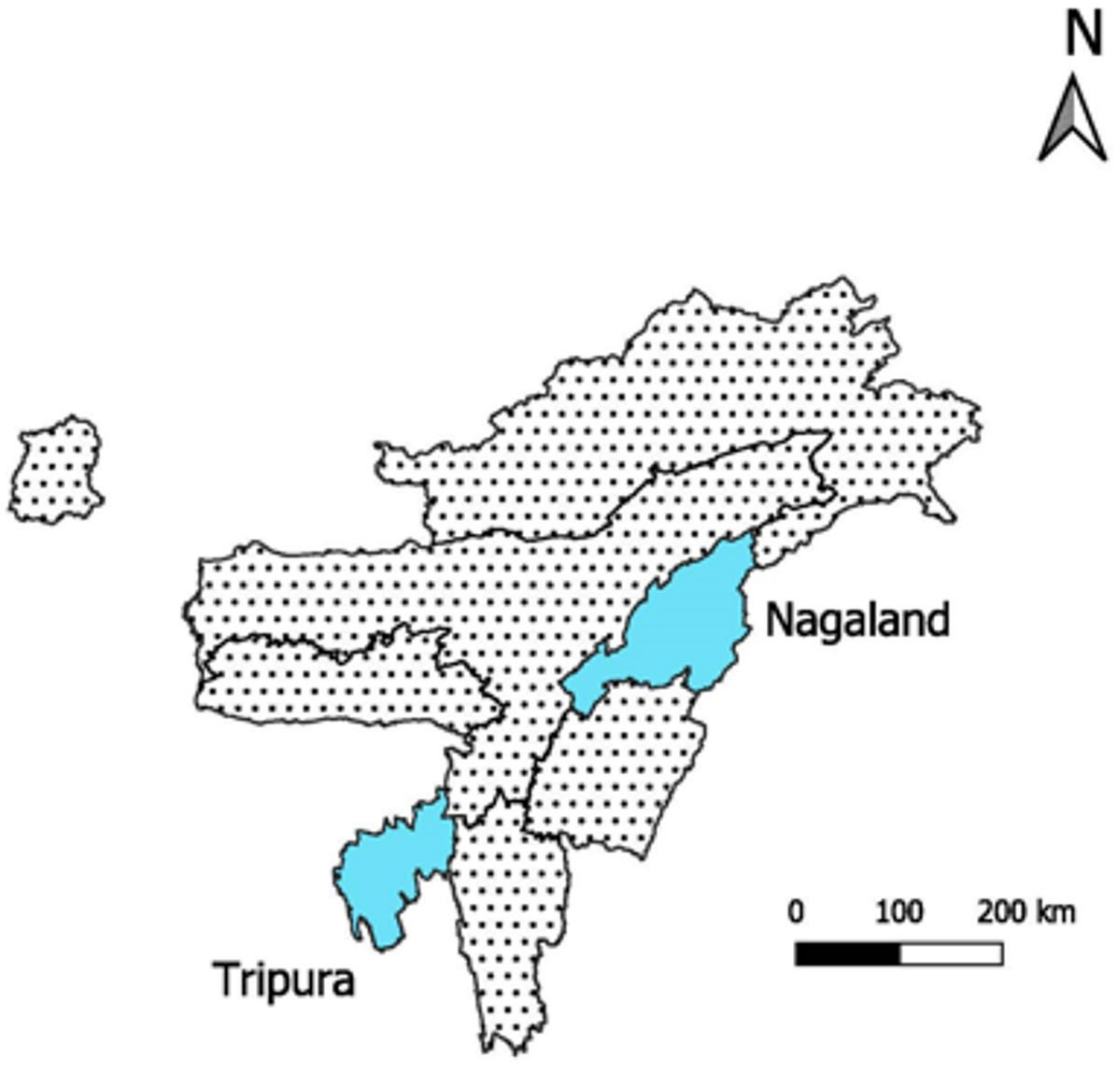
Map of Northeastern states of India showing the two study states Nagaland and Tripura coloured blue. The map was created by the authors using QGIS Geographic Information System version (3.44.9-Solothurn; QGIS Association; https://www.qgis.org) using India ADM1 administrative boundary data from geoBoundaries. [Runfola D, et al. 2020. geoBoundaries: A global database of political administrative boundaries. PLOS ONE 15(4): e0231866. 10.1371/journal.pone.0231866]. QGIS.org. 2026. QGIS Geographic Information System. QGIS Association.

A case–control study was conducted in the two northeastern Indian states of Nagaland and Tripura (Fig. 1) after approval by the Institutional Ethics Committee/Institutional Review Board of the ICMR–Regional Medical Research Centre, N.E. Region, Dibrugarh, Assam (Approval No. RMRC/Dib/IEC (Human)/2013-14/181). Written informed consent was obtained from all participants prior to enrolment. The study included 190 newly diagnosed gastric cancer patients recruited from two hospitals (one in Nagaland and one in Tripura) and 317 apparently healthy controls. The controls were community-based neighbourhood subjects, not hospital visitors, and were enrolled from the same catchment areas as the cases.

### Inclusion and exclusion criteria for cases

Inclusion criteria: Primary, new cases of gastric carcinoma confirmed by histopathology. Exclusion criteria: Metastatic disease obscuring the primary site, recurrent gastric cancer cases, Cases unable to provide reliable information (e.g., severe debility, cognitive impairment), cases refusing to participate, and cases who underwent blood transfusion (≤ 6 months before enrolment).

### Controls

The controls were community-based neighbourhood subjects, not hospital visitors, and were enrolled from the same catchment areas as the cases.

### Questionnaire and exposure assessment

Trained interviewers administered a pre-coded questionnaire covering sociodemographic variables, tobacco and betel-nut habits (smoking, chewing); categorised as *ever* vs. *never* users. Dietary intake of vegetables, fruits, was recorded with a six-month recall period.

### Sample collection

From each participant, 3–5 mL of whole blood was collected in EDTA-coated vials for DNA extraction from peripheral blood mononuclear cells (PBMCs). In addition, 5 mL of blood was collected to obtain serum for *Helicobacter pylori* serological analysis. All specimens were stored at − 80 °C until analysis.

### Genotyping assays

Detoxification genes: GSTM1, GSTT1, and CYP2E1 polymorphisms were typed by PCR^24^. Innate-immunity genes TLR2 and TLR4 were typed by PCR-RFLP^6,18,25^. Cytokine genes: *IL-10* and *TNF-α* variants were genotyped by PCR–RFLP^26,27^.

### Germline-mutation profiling

Mutations in key gastric-cancer genes (APC, BRAF, CDH1, CDKN2A, CTNNB1, ERBB2, FBXW7, HRAS, KRAS, NRAS, PDGFRA, PIK3CA, and TP53) were interrogated in 80 PMBC samples from gastric cancer patients (Tripura *n* = 39; Nagaland *n* = 41) using the qBiomarker™ Human Gastric Cancer Somatic Mutation PCR Array (Qiagen). This mutation Realtime PCR Assays use allele-specific amplification and probe-based detection to identify mutations present in as little as 1% of a wild-type DNA background. This is achieved using ARMS^®^ technology, which relies on Taq polymerase’s ability to distinguish mismatches at the 3′ end of PCR primers. We performed mutation-panel screening in a subset of cases (*n* = 80). This was due to economic/resource constraints. The subset was selected to maintain representation from both states.

### Helicobacter pylori serology

Serum IgG antibodies against *Helicobacter pylori* were detected by ELISA as described previously^28^.

### Dietary factors

Dietary intake of selected foods (pumpkin, cabbage, cauliflower, tomato, other vegetables, and fruits) was assessed among cases and controls through face-to-face interviews. Information was recorded using a pre-designed food-frequency questionnaire (FFQ) to evaluate the potential protective or detrimental effects of these foods.

### Statistical analysis

Data were entered in IBM SPSS Statistical software v26 with routine quality checks. Descriptive statistics summarised participant characteristics. Univariate binary logistic regression estimated crude odds ratios (ORs) and 95% confidence intervals (CIs). Multivariable logistic regression, adjusted for age, sex, and state, was undertaken to get adjusted ORs to identify independent risk factors. A priori power calculations indicated that 150 cases and 150 controls would detect an OR = 2.0 for an exposure prevalence of 25% at 80% power (α = 0.05); All P-values are two-sided; *p* < 0.05 was considered statistically significant.

## Results

### Demographic characteristics of gastric cancer cases and controls, and histopathology of gastric tumours

A total of 190 gastric cancer cases and 317 apparently healthy community-based neighbourhood controls were included in the study. The mean age of the gastric cancer patients was 52.9 ± 14.5 years. Among cases, 14.7% were aged ≤ 40 years, 46.3% were aged 41–60 years, and 38.9% were aged ≥ 61 years. In the control group, the corresponding proportions were 24.0%, 42.9%, and 33.1%, respectively. Men predominated in both groups, accounting for 67.4% of cases and 64.4% of controls, while women represented 32.6% of cases and 35.6% of controls. Additional demographic characteristics of cases and controls, including educational status, occupational status, family history of cancer, and smoking status, are summarized in Supplementary Table S1. Histopathology reports were available for 161 of 190 gastric cancer cases (84.7%), and Lauren classification was extracted from the original pathology reports issued by certified pathologists at the two participating hospitals. Among these 161 gastric adenocarcinoma cases, the intestinal type predominated (106/161, 65.8%), followed by mixed (21/161, 13.0%) and diffuse (16/161, 9.9%). Lauren subtype could not be assigned from the available reports for 18 cases (11.2%), which were categorized as unclassifiable/other (Supplementary Table S2).

### Genetic polymorphisms in *GSTT1*, *GSTM1*, and *CYP2E1*

The associations of *GSTT1*, *GSTM1*, and *CYP2E1* polymorphisms with gastric cancer were evaluated using univariate logistic regression and multivariable logistic regression adjusted for age, sex, and state (Table 1). The *GSTT1-*null genotype was present in 32.6% of cases and 30.9% of controls and was not significantly associated with gastric cancer in either univariate analysis (OR 1.08, 95% CI 0.74–1.59; *p* = 0.687) or adjusted analysis (adjusted OR 1.29, 95% CI 0.82–2.03; *p* = 0.266). In contrast, the *GSTM1-*null genotype was significantly more frequent among cases than controls (54.2% vs. 39.4%) and was associated with increased gastric cancer risk in both univariate (OR 1.82, 95% CI 1.26–2.62; *p* = 0.001) and adjusted analyses (adjusted OR 2.26, 95% CI 1.49–3.43; *p* < 0.001). For *CYP2E1*, the *C1/C2* genotype was detected in 9.5% of cases and 5.7% of controls. Although the univariate association was not significant (OR 1.69, 95% CI 0.86–3.40; *p* = 0.130), the association became significant after adjustment (adjusted OR 2.24, 95% CI 1.03–4.87; *p* = 0.042). The *C2/C2* genotype was observed only among controls.

**Table 1:** Genetic polymorphism of *GSTT1*, *GSTM1* and *CYP2E1* and risk of gastric cancer based on Logistic regression analysis. Gastric cancer cases are from Nagaland and Tripura and apparently health controls are from the community (neighbourhood of cases).

Factors	Case (*n* = 190)	Control (*n* = 317)	Univariate logistic regression		Multiple logistic regression	
	*n* (%)	*n* (%)	OR (95% CI)	*p*-value	OR (95% CI)	*p*-value
*GSTT1*						
Non-Null	128 (67.4)	219 (69.1)	1		1	
Null	62 (32.6)	98 (30.9)	1.08 (0.74–1.59)	0.687	1.29 (0.82–2.03)	0.266
*GSTM1*						
Non-Null	87 (45.8)	192 (60.6)	1		1	
Null	103 (54.2)	125 (39.4)	1.82 (1.26–2.62)	0.001*	2.26 (1.49–3.43)	< 0.001*
*CYP2E1*						
C1/C1	172 (90.5)	291 (91.8)	1		1	
C1/C2	18 (9.5)	18 (5.7)	1.69 (0.86–3.40)	0.130	2.24 (1.03–4.87)	0.042*
C2/C2	0 (0.0)	8 (2.5)	--		--	

Adjusted for age, sex and state in multiple logistic regression model.

*Significant P value.

### Tobacco and betel-nut chewing

Tobacco chewing was reported by 25.8% of gastric cancer cases compared with 8.8% of controls and was significantly associated with increased gastric cancer risk in both univariate (OR 3.59, 95% CI 2.16–5.95; *p* < 0.001) and adjusted analyses (adjusted OR 2.71, 95% CI 1.52–4.83; *p* = 0.001). Betel-nut chewing was also more frequent among cases than controls (55.3% vs. 27.4%) and remained significantly associated with gastric cancer in both univariate (OR 3.27, 95% CI 2.24–4.76; *p* < 0.001) and adjusted analyses (adjusted OR 2.24, 95% CI 1.45–3.48; *p* < 0.001) (Supplementary Table S3).

### Interaction of *GSTT1* with tobacco and betel-nut chewing

No statistically significant interaction was observed between the *GSTT1*-null genotype and chewing habits after adjustment. Among never tobacco chewers, *GSTT1*-null genotype was not associated with gastric cancer (adjusted OR 1.12, 95% CI 0.70–1.81; *p* = 0.627), while among ever tobacco chewers the risk estimate was higher but remained non-significant (adjusted OR 2.81, 95% CI 0.82–9.58; *p* = 0.099). A similar pattern was seen for betel-nut chewing. Among never betel-nut chewers, *GSTT1*-null genotype was not associated with risk (adjusted OR 1.21, 95% CI 0.69–2.12; *p* = 0.508), while among ever betel-nut chewers the crude association was significant but lost significance after adjustment (adjusted OR 1.53, 95% CI 0.73–3.19; *p* = 0.256) (Supplementary Tables S4 and S5).

### Interaction of *GSTM1* with tobacco and betel-nut chewing

The association between the *GSTM1*-null genotype and gastric cancer remained significant in the stratified analyses and was strongest among tobacco chewers. Among never tobacco chewers, *GSTM1*-null genotype was associated with increased gastric cancer risk after adjustment (adjusted OR 1.76, 95% CI 1.14–2.70; *p* = 0.011). Among ever tobacco chewers, individuals carrying the *GSTM1*-null genotype had a markedly higher risk than tobacco chewers carrying the *GSTM1* non-null genotype (adjusted OR 12.63, 95% CI 3.04–52.42; *p* < 0.001) (Tables 2, 3, 4). A similar pattern was observed for betel-nut chewing. *GSTM1*-null genotype was associated with increased gastric cancer risk among never betel-nut chewers (adjusted OR 2.22, 95% CI 1.29–3.82; *p* = 0.004) and ever betel-nut chewers (adjusted OR 2.57, 95% CI 1.34–4.92; *p* = 0.004) (Supplementary Table S6).

**Table 2:** Interaction of *GSTM1* polymorphism and tobacco chewing habit and risk of Gastric cancer.

*GSTM1* and tobacco chewing habits		Case	Control	Univariate logistic regression		Multiple logistic regression	
		n (%)	n (%)	OR (95% CI)	p-value	OR (95% CI)	p-value
Never chewer	Non-null	68 (48.2)	168 (58.1)	1		1	
	Null	73 (51.8)	121 (41.9)	1.49 (0.99–2.23)	0.053*	1.76 (1.14–2.70)	0.011*
Ever chewer	Non-null	19 (38.8)	24 (85.7)	1		1	
	Null	30 (61.2)	4 (14.3)	9.47 (2.84–31.59)	< 0.001*	12.63 (3.04–52.42)	< 0.001*

Adjusted for age, sex and state in multiple logistic regression model.

*Significant P value.

**Table 3:** Prevalence of germline mutations in a cohort of 80 Gastric Cancer patients from Tripura and Nagaland.

Genes	Gene Description	Function	Total Mutation Count	Mut.Freq %	Number of Samples where mutation is present
*TP53*	Tumor protein p53	Tumor Suppressor Gene	138	81.25	65
*CTNNB1*	Catenin Beta 1 (alias cadherin associated protein beta 1)	Oncogene (Beta catenin pathway)	118	71.25	57
*APC*	Adenomatous polyposis coli	Tumor Suppressor Gene	51	53.75	43
*CDKN2A*	Cyclin dependent kinase inhibitor 2 A	Tumor Suppressor Gene	25	31.25	25
*KRAS*	Kirsten rat sarcoma viral oncogene homolog	Oncogene	16	12.5	10
*PIK3CA*	Phosphatidylinositol-4,5-bisphosphate 3-kinase catalytic subunit?	Oncogene	9	10	8
*ERBB2*	Receptor tyrosine kinase 2 (HER2/neu)	Oncogene	5	6.25	5
*PDGFRA*	Platelet-derived growth factor receptor alpha	Oncogene	3	3.75	3
*BRAF*	B-Raf proto-oncogene, serine/threonine kinase	Oncogene	1	1.25	1
*CDH1*	Cadherin 1	Tumor Suppressor Gene	1	1.25	1
*FBXW7*	F-box and WD repeat domain-containing protein 7	Tumor Suppressor Gene	1	1.25	1
*HRAS*	Harvey rat sarcoma viral oncogene homolog	Oncogene	1	1.25	1
*NRAS*	Neuroblastoma RAS viral oncogene homolog	Oncogene	1	1.25	1

**Table 4:** Frequency of germline missense and truncating mutations in tumor suppressor genes and oncogenes in 80 gastric cancer patients from Nagaland and Tripura (Detected by real-time PCR assays for assessing the status of validated mutations within a pathway-focused set of genes (Qaigen)).

Genes and their mutations	COSMIC ID AA change	rsID	ClinVar category	Tripura (*n* = 39)	Nagaland (*n* = 41)	Total (*n* = 80)
*APC*						
c.3927_3931delAAAGA	13,113 p.E1309fs*4	rs121913224	Pathogenic	2 (5.1%)	1 (2.4%)	3 (3.8%)
c.4348 C > T	13,127 p.R1450*	rs121913332	Pathogenic	18 (46.2%)	20 (48.8%)	38 (47.5%)
c.4391_4394delAGAG	18,838 p.E1464fs*8	rs387906234	Pathogenic	1 (2.6%)	0	1 (1.2%)
c.4666_4667insA	18,561 p.T1556fs*3	rs587783031	Pathogenic	2 (5.1%)	7 (17.1%)	9 (11.2%)
*BRAF*						
c.1798G > A	1130 p.V600M	rs121913378	Likely pathogenic	0	0	0
c.1799T > A	476 p.V600E	rs113488022	Pathogenic	1 (2.6%)	0	1 (1.2%)
*CDH1*						
c.1108G > C	19,748 p.D370H	rs1960859184	Uncertain significance	1 (2.6%)	0	1 (1.2%)
*CDKN2A*						
c.238 C > T	12,475 p.R80*	rs121913388	Pathogenic/Likely pathogenic	14 (35.9%)	11 (26.8%)	25 (31.2%)
*CTNNB1*						
c.101G > A	5671 p.G34E	rs28931589*	Pathogenic	0	0	0
c.101G > T	5670 p.G34V	rs28931589*	Likely pathogenic	0	0	0
c.106 C > T	5703 p.H36Y	No rsID found**	Not listed	9 (23.1%)	6 (14.6%)	15 (18.8%)
c.109T > G	5675 p.S37A	rs121913228	Likely pathogenic	2 (5.1%)	1 (2.4%)	3 (3.8%)
c.110 C > A	5666 p.S37Y	rs121913403	Likely pathogenic	0	1 (2.4%)	1 (1.2%)
c.110 C > G	5679 p.S37C	rs121913403	Likely pathogenic	6 (15.4%)	8 (19.5%)	14 (17.5%)
c.110 C > T	5662 p.S37F	rs121913403	Pathogenic	19 (48.7%)	12 (29.3%)	31 (38.8%)
c.112_114GGT > CCC	24,746 p.G38P	No rsID found	Not listed	3 (7.7%)	1 (2.4%)	4 (5.0%)
c.143G > A	5733 p.G48D	No rsID found	Not listed	10 (25.6%)	11 (26.8%)	21 (26.2%)
c.130 C > G	17,661 p.P44A	No rsID found	Not listed	2 (5.1%)	0	2 (2.5%)
c.86 C > T	5694 p.S29F	rs1443348691	Uncertain significance	5 (12.8%)	3 (7.3%)	8 (10.0%)
c.94G > A	5672 p.D32N	rs28931588	Pathogenic	1 (2.6%)	1 (2.4%)	2 (2.5%)
c.94G > C	5668 p.D32H	rs28931588	Not listed	0	0	0
c.94G > T	5661 p.D32Y	rs28931588	Pathogenic	8 (20.5%)	5 (12.2%)	13 (16.2%)
c.95 A > G	5681 p.D32G	rs121913396	Likely pathogenic	2 (5.1%)	1 (2.4%)	3 (3.8%)
c.98 C > A	5673 p.S33Y	rs121913400	Conflicting Interpretations “Other”	0	0	0
c.98 C > G	5677 p.S33C	rs121913400	Pathogenic	0	1 (2.4%)	1 (1.2%)
c.98 C > T	5669 p.S33F	rs121913400	Pathogenic	0	0	0
*ERBB2*						
c.2326G > A	685 p.G776S	rs28933369	Likely pathogenic	0	0	0
c.2329G > T	14,062 p.V777L	rs121913471	VUS	2 (5.1%)	3 (7.3%)	5 (6.2%)
*FBXW7*						
c.1393 C > T	22,932 p.R465C	rs867384286	Pathogenic	1 (2.6%)	0	1 (1.2%)
*HRAS*						
c.35G > T	483 p.G12V	rs121913529	Pathogenic	0	1 (2.4%)	1 (1.2%)
*KRAS*						
c.175G > A	546 p.A59T	rs121913528	Pathogenic	2 (5.1%)	1 (2.4%)	3 (3.8%)
c.182 A > G	552 p.Q61R	rs121913240	Conflicting Interpretations	0	0	0
c.34G > A	517 p.G12S	rs121913530	Pathogenic	2 (5.1%)	0	2 (2.5%)
c.34G > T	516 p.G12C	rs121913530	Likely Pathogenic	1 (2.6%)	2 (4.9%)	3 (3.8%)
c.35G > A	521 p.G12D	rs121913529	Pathogenic/Likely Pathogenic	0	0	0
c.35G > C	522 p.G12A	rs121913529	Pathogenic/Likely Pathogenic	1 (2.6%)	1 (2.4%)	2 (2.5%)
c.35G > T	520 p.G12V	rs121913529	Pathogenic	3 (7.7%)	2 (4.9%)	5 (6.2%)
c.37G > A	528 p.G13S	rs121913535	Pathogenic	0	0	0
c.37G > T	527 p.G13C	rs121913535	Pathogenic	0	0	0
c.38G > A	532 p.G13D	rs112445441	Pathogenic	0	1 (2.4%)	1 (1.2%)
*NRAS*						
c.37G > C	569 p.G13R	rs121434595	Likely Pathogenic	0	1 (2.4%)	1 (1.2%)
*PDGFRA*						
c.1698_1712del15	12,418 p.S566_E571 > R	rs2110299748	Not listed	2 (5.1%)	1 (2.4%)	3 (3.8%)
*PIK3CA*						
c.1624G > A	760 p.E542K	rs121913273	Pathogenic	3 (7.7%)	3 (7.3%)	6 (7.5%)
c.1633G > A	763 p.E545K	rs104886003	Pathogenic/Likely pathogenic	0	1 (2.4%)	1 (1.2%)
c.3062 A > G	12,461 p.Y1021C	rs121913288	Pathogenic	0	0	0
c.3140 A > G	775 p.H1047R	rs121913279	Pathogenic	1 (2.6%)	1 (2.4%)	2 (2.5%)
*TP53*						
c.404G > T	10,647 p.C135F	rs587781991	Pathogenic/Likely pathogenic	2 (5.1%)	3 (7.3%)	5 (6.2%)
c.422G > A	43,708 p.C141Y	rs587781288	Pathogenic/Likely pathogenic	0	1 (2.4%)	1 (1.2%)
c.451 C > T	10,905 p.P151S	rs28934874	Likely pathogenic	4 (10.3%)	10 (24.4%)	14 (17.5%)
c.461G > T	6815 p.G154V	rs762846821	Conflicting Interpretations	1 (2.6%)	1 (2.4%)	2 (2.5%)
c.469G > T	10,670 p.V157F	rs121912654	Likely pathogenic	4 (10.3%)	2 (4.9%)	6 (7.5%)
c.473G > A	10,690 p.R158H	rs587782144	Likely pathogenic	15 (38.5%)	12 (29.3%)	27 (33.8%)
c.476 C > T	11,148 p.A159V	rs1555526131	Pathogenic/Likely pathogenic	2 (5.1%)	0	2 (2.5%)
c.481G > A	10,739 p.A161T	rs193920817	Pathogenic/Likely pathogenic	0	0	0
c.488 A > G	10,808 p.Y163C	rs148924904	Pathogenic	1 (2.6%)	0	1 (1.2%)
c.517G > T	43,559 p.V173L	rs876660754	Pathogenic/Likely pathogenic	1 (2.6%)	0	1 (1.2%)
c.524G > A	10,648 p.R175H	rs28934578	Pathogenic	1 (2.6%)	1 (2.4%)	2 (2.5%)
c.527G > T	10,645 p.C176F	rs786202962	Pathogenic/Likely pathogenic	2 (5.1%)	0	2 (2.5%)
c.574 C > T	10,733 p.Q192*	rs866380588	Pathogenic	10 (25.6%)	6 (14.6%)	16 (20.0%)
c.578 A > G	10,742 p.H193R	rs786201838	Pathogenic/Likely pathogenic	2 (5.1%)	1 (2.4%)	3 (3.8%)
c.586 C > T	10,705 p.R196*	rs397516435	Pathogenic	0	0	0
c.637 C > T	10,654 p.R213*	rs397516436	Pathogenic	0	0	0
c.659 A > G	10,758 p.Y220C	rs121912666	Pathogenic	0	0	0
c.707 A > G	10,731 p.Y236C	rs730882026	Pathogenic/Likely pathogenic	2 (5.1%)	1 (2.4%)	3 (3.8%)
c.711G > A	10,834 p.M237I	rs587782664	Pathogenic	4 (10.3%)	8 (19.5%)	12 (15.0%)
c.730G > A	10,941 p.G244S	rs1057519989	Pathogenic	8 (20.5%)	8 (19.5%)	16 (20.0%)
c.733G > A	6932 p.G245S	rs28934575	Pathogenic	2 (5.1%)	2 (4.9%)	4 (5.0%)
c.734G > A	43,606 p.G245D	rs121912656	Pathogenic	1 (2.6%)	2 (4.9%)	3 (3.8%)
c.742 C > T	10,656 p.R248W	rs121912651	Pathogenic	0	0	0
c.743G > A	10,662 p.R248Q	rs11540652	Pathogenic	2 (5.1%)	1 (2.4%)	3 (3.8%)
c.746G > T	43,871 p.R249M	rs587782329	VUS	0	2 (4.9%)	2 (2.5%)
c.797G > A	10,867 p.G266E	rs193920774	Likely pathogenic	0	1 (2.4%)	1 (1.2%)
c.808T > C	44,262 p.F270L	rs1057519988	Conflicting Interpretations	0	0	0
c.817 C > T	10,659 p.R273C	rs121913343	Pathogenic/Likely pathogenic	0	0	0
c.818G > A	10,660 p.R273H	rs28934576	Pathogenic	0	0	0
c.824G > A	10,893 p.C275Y	rs863224451	Likely pathogenic	4 (10.3%)	3 (7.3%)	7 (8.8%)
c.832 C > T	10,939 p.P278S	rs17849781	Pathogenic/Likely pathogenic	0	0	0
c.833 C > G	10,887 p.P278R	rs876659802	Conflicting Interpretations	2 (5.1%)	0	2 (2.5%)
c.844 C > G	10,992 p.R282G	rs28934574	Pathogenic	1 (2.6%)	1 (2.4%)	2 (2.5%)
c.844 C > T	10,704 p.R282W	rs28934574	Pathogenic	0	1 (2.4%)	1 (1.2%)
c.853G > A	10,722 p.E285K	rs112431538	Pathogenic/Likely pathogenic	0	0	0

*This rs site has multiple alternate alleles.

** This variant is reported as a **somatic** *CTNNB1* hotspot alteration in cancer knowledgebases/literature rather than as a curated germline ClinVar entry.

### Interaction of *CYP2E1* with tobacco and betel-nut chewing

The *CYP2E1* C1/C2 genotype showed elevated odds ratios in the chewing-stratified analyses, but none of these associations reached statistical significance after adjustment. Among never tobacco chewers, the adjusted OR was 2.03 (95% CI 0.89–4.66; *p* = 0.094), whereas among ever tobacco chewers it was 5.11 (95% CI 0.46–56.81; *p* = 0.184). Among never betel-nut chewers, the adjusted OR was 2.43 (95% CI 0.97–6.06; *p* = 0.057), while among ever betel-nut chewers it was 3.37 (95% CI 0.60–19.07; *p* = 0.168). These wide confidence intervals indicate limited precision due to small numbers within strata (Supplementary Tables S7 and S8).

### *TLR2*, *TLR4*, *TNF*-α, and *IL-10* polymorphisms

No significant association was observed for *TLR2* and *TLR4* polymorphisms in either univariate or age-, sex-, and state-adjusted logistic regression analyses. Likewise, no significant association was found for the *TNF-α* and *IL-10-*592 polymorphisms (Supplementary Table S9).

### Helicobacter pylori seroprevalence

Among healthy community controls, *Helicobacter pylori* seropositivity was higher in Tripura than in Nagaland (79.3% vs. 58.3%). Within Tripura, seropositivity was higher in males than in females (82.1% vs. 72.5%). Despite this high background prevalence, *H. pylori* seropositivity was not significantly associated with gastric cancer risk.

### Dietary factors

Regular consumption of pumpkin, seasonal fruits, tomato, cabbage, and cauliflower was associated with reduced gastric cancer risk. After adjustment for age, sex, state, tobacco chewing, and betel-nut chewing, the protective association remained strong (Supplementary Table S10).

### Germline mutation profiling

Exploratory mutation profiling was performed on germline DNA from 80 gastric cancer patients (Tripura, *n* = 39; Nagaland, *n* = 41) using the Human Gastric Cancer qBiomarker Somatic Mutation PCR Array, which was applied here to screen for putative germline alterations in genes commonly implicated in gastric carcinogenesis (Table 3).

Among the genes screened, *TP53* was the most frequently altered gene, with mutations detected in 65/80 patients (81.25%), followed by *CTNNB1* in 57/80 (71.25%), *APC* in 43/80 (53.75%), and *CDKN2A* in 25/80 (31.25%) (Table 3). Detailed frequencies of individual variants, together with COSMIC ID, rsID, and ClinVar category, are shown in Table 4.

The supplementary table S11 provides the detailed patient wise data (*n* = 80). In this mutation profiled subset of gastric cancer patient, age ranged from 27 to 84 years (mean 56.4 years). The overall mutation burden ranged from 0 to 24 mutant calls per patient (mean 4.6, median 4). Only 3/80 patients had no detected mutation, whereas 71/80 (88.8%) had two or more mutations and 37/80 had five or more mutations.

Age-stratified analysis (supplementary table S12) showed that the young group (< 40 years; *n* = 11) had the highest mutation burden (72 total mutations; mean 6.55 mutations/patient) compared with the 40–59 year group (129 total mutations; mean 3.91 mutations/patient) and the ≥ 60 year group (169 total mutations; mean 4.69 mutations/patient). One young patient (TR-39, age 30 years) had the highest individual burden, with 24 mutations. *TP53* and *CTNNB1* were the most frequently altered genes across all age groups and were particularly common among younger patients. A relative enrichment in younger cases was also observed for *CDKN2A*, and to a lesser extent for *ERBB2* and *PIK3CA*. Because the younger subgroup was small, these findings should be interpreted cautiously.

## Discussion

Gastric cancer remains an important public health problem in Northeast India, as reflected in population-based cancer registry data, and variations in cancer incidence and outcome across ethnic and geographic groups may partly reflect differences in inherited and acquired genetic factors as well as lifestyle and environmental exposures^29–31^. In this context, the present study provides epidemiological and molecular data from patients in Nagaland and Tripura, two populations that have been underrepresented in gastric cancer research.

The demographic profile of our patients was broadly consistent with previous Indian reports, with a predominance of males and a high frequency of cases in the 41–60-year age group^31^. Lauren classification was available for 161 of 190 cases, and the intestinal subtype predominated (65.8%), followed by mixed (13.0%) and diffuse (9.9%) (Supplementary Table S1). This distribution suggests that intestinal-pattern gastric adenocarcinoma was the most common histological subtype in the present cohort.

A major finding of the study was the significant association of the *GSTM1* null genotype with gastric cancer, both overall and in stratified analyses. The risk was especially pronounced among tobacco chewers carrying the *GSTM1* null genotype, supporting a strong gene–environment interaction. Because *GSTM1* is involved in detoxification of carcinogenic intermediates, deletion of this gene may reduce the ability to clear tobacco-related mutagens and thereby enhance susceptibility to gastric carcinogenesis. Similar observations have been reported previously in gastric and gastrointestinal cancer studies^3,15,32^. In contrast, *GSTT1* showed no significant overall association, and the evidence for interaction with chewing habits was weaker. The *CYP2E1* C1/C2 genotype became significant only after adjustment in the overall model, while stratified analyses yielded elevated but non-significant odds ratios with wide confidence intervals, suggesting the need for larger studies.

Both tobacco chewing and betel-nut chewing were independently associated with gastric cancer in this study. These findings are biologically plausible, since smokeless tobacco and betel nut contain carcinogenic compounds capable of causing chronic mucosal injury, oxidative stress, and inflammation^33,34^. They are also consistent with previous findings from high-risk populations in Northeast India^3–7^.

Another important component of the study was the exploratory profiling of germline DNA using the qBiomarker mutation PCR array. Although this platform was originally developed for detection of somatic hotspot mutations in tumour tissue, it was applied here to germline DNA from 80 gastric cancer patients to screen for putative inherited alterations in genes commonly implicated in gastric carcinogenesis. In this subset, only 3 of 80 patients had no detected mutation, whereas 71 of 80 had two or more mutations and 37 had five or more mutations. The overall mutation burden ranged from 0 to 24 mutations per patient, and the younger subgroup showed the highest mean mutation burden per patient, although the number of younger patients was small (Supplementary Table S11).

Within this exploratory germline analysis, TP53 emerged as the most frequently altered gene, followed by *CTNNB1*, *APC*, and *CDKN2A* (Table 2). The prominence of *TP53* is noteworthy because germline *TP53* mutations are a hallmark of Li-Fraumeni syndrome^35–37^. The frequent alterations observed in *CTNNB1* and *APC* suggest possible involvement of the Wnt/β-catenin pathway, which is an established pathway in cancer development^38^. Detection of *CDH1* alterations is also of interest, because pathogenic germline *CDH1* variants are central to hereditary diffuse gastric cancer^39^.

The observation that many patients carried multiple putative germline alterations, together with the relatively higher mutation burden in the younger subgroup, raises the possibility that inherited susceptibility may contribute to gastric cancer in at least a subset of cases. This interpretation is also indirectly supported by the family-history data in the broader cohort: 21 of 190 patients (11.1%) reported a family history of cancer, and the mean age at diagnosis was lower in these patients than in those without such history (51.5 vs. 57.2 years), although the difference did not reach statistical significance (*p* = 0.07). These findings suggest a possible trend toward earlier onset in patients with familial cancer aggregation, but the present study was not designed as a formal hereditary cancer work-up and therefore cannot establish hereditary syndromic classification.

An additional point requiring careful interpretation is the detection in germline DNA of variants that are more commonly discussed as somatic hotspot alterations, including changes in *BRAF*, *KRAS*, *NRAS*, and *PIK3CA*. These genes participate in key oncogenic pathways such as *RAS/RAF/MEK/ERK* and *PI3K/AKT* signalling^40^. Their detection in the present study is intriguing, but such findings should not be overinterpreted as definitive evidence of inherited predisposition and need further comprehensive studies. The mutation platform used in this study has been evaluated previously against sequencing-based methods. In a comparative validation study, whole-exome sequencing confirmed 65 of 71 (92%) qBiomarker-detected substitutions that were tested supporting the reliability of this allele-specific real-time PCR approach for predefined hotspot mutation detection^41^. This supports the platform’s analytical utility.

In contrast to the findings for xenobiotic-metabolizing genes, polymorphisms in *TLR2*, *TLR4*, *TNF*-α, and *IL-10* were not significantly associated with gastric cancer in our study. This may reflect limited statistical power, population-specific effects, or the broader complexity of inflammation-related susceptibility in gastric carcinogenesis. Similarly, although *Helicobacter pylori* is a well-established gastric cancer risk factor, seropositivity was not significantly associated with gastric cancer in our study. This may partly reflect the high background prevalence of infection in the community and the known limitations of serological testing, which does not distinguish current infection from past exposure^17,20,25,42^.

Our findings also support a protective role of diet. Regular consumption of vegetables and fruits such as pumpkin, seasonal fruits, tomato, cabbage, and cauliflower was associated with reduced gastric cancer risk. These observations are consistent with the broader concept that diet may influence gastric carcinogenesis and suggest that dietary modification may be an important component of prevention strategies in this region^43^.

Several limitations should be considered while interpreting the findings. First, germline mutation profiling was restricted to an exploratory subset of 80 cases because of cost constraints and the need to ensure representation from both Tripura and Nagaland. Second, formal hereditary cancer evaluation, detailed pedigree analysis, and study of relatives were not undertaken because this was an exploratory/pilot investigation. These issues should be addressed in future studies through larger cohorts, NGS sequencing, clinical genetic assessment, and family-based analyses.

## Conclusion

In conclusion, this study provides new epidemiological and molecular insights into gastric cancer in the understudied populations of Nagaland and Tripura. The findings highlight the importance of gene–environment interactions, particularly the association of the *GSTM1* null genotype with gastric cancer risk and its markedly stronger effect among tobacco chewers. The study also suggests that a subset of patients may carry a relatively high burden of putative germline alterations, particularly involving *TP53* and genes related to the Wnt signalling pathway, such as *CTNNB1* and *APC*. To draw definitive conclusions regarding hereditary cancer syndromes, family-based risk assessment, or immediate clinical application need to be undertaken in cancer patients from NE region of India. Overall, the study provides an initial framework for future research on gastric cancer susceptibility in Northeast India. Larger studies integrating confirmatory germline and somatic mutation sequencing, pedigree assessment, family history, lifestyle exposures, diet, and microbial factors will be important for clarifying the contribution of inherited predisposition and gene–environment interactions in these populations. Such work may ultimately help improve regional risk stratification, prevention strategies, and clinical management.

## Supplementary Information

Below is the link to the electronic supplementary material.

Supplementary Material 1

Supplementary Material 2

Supplementary Material 3

Supplementary Material 4

Supplementary Material 5

Supplementary Material 6

Supplementary Material 7

Supplementary Material 8

Supplementary Material 9

Supplementary Material 10

Supplementary Material 11

Supplementary Material 12

## Data Availability

The datasets used and/or analysed during the current study are available from the corresponding author on reasonable request.

## References

[1] Singh, K., Grover, A. & Dhanasekaran, K. Unveiling the cancer epidemic in India: a glimpse into GLOBOCAN 2022 and past patterns Singh, Khushwant. *Lancet Reg. Health - Southeast. Asia Volume*. **34**, 34 (2025). 10.1016/j.lansea.2025.100546 PMC11893321 40070552

[2] National Centre for Disease Informatics and Research (NCDIR), N. C. R. P., Indian Council of Medical Research (ICMR). *Report of National Cancer Registry Programme (2012–2016)* (ICMR–NCDIR, 2020).

[3] Malakar, M. et al. Genetic polymorphism of glutathione S-transferases M1 and T1, tobacco habits and risk of stomach cancer in Mizoram, India. *Asian Pac. J. Cancer Prev.* **13**, 4725–4732. 10.7314/apjcp.2012.13.9.4725 (2012). 23167410 10.7314/apjcp.2012.13.9.4725

[4] Malakar, M. et al. CYP2E1 genetic polymorphism with dietary, tobacco, alcohol habits, *H. pylori* infection status and susceptibility to stomach cancer in Mizoram, India. *Asian Pac. J. Cancer Prev.* **15**, 8815–8822. 10.7314/apjcp.2014.15.20.8815 (2014). 25374213 10.7314/apjcp.2014.15.20.8815

[5] Malakar, M. et al. p53 codon 72 polymorphism interactions with dietary and tobacco related habits and risk of stomach cancer in Mizoram, India. *Asian Pac. J. Cancer Prev.* **15**, 717–723. 10.7314/apjcp.2014.15.2.717 (2014). 24568485 10.7314/apjcp.2014.15.2.717

[6] Mukherjee, D. et al. Association of toll-like receptor 2 ∆22 and risk for gastric cancer considering main effects and interactions with smoking: A matched case-control study from Mizoram, India. *Tumour Biol.* **37**, 10821–10826. 10.1007/s13277-016-4982-5 (2016). 26880585 10.1007/s13277-016-4982-5

[7] Phukan, R. K., Zomawia, E., Narain, K., Hazarika, N. C. & Mahanta, J. Tobacco use and stomach cancer in Mizoram, India. *Cancer Epidemiol. Biomarkers Prev.* **14**, 1892–1896. 10.1158/1055-9965.EPI-05-0074 (2005). 16103433 10.1158/1055-9965.EPI-05-0074

[8] Ghatak, S. et al. Xenobiotic pathway gene polymorphisms associated with gastric cancer in high risk Mizo-Mongoloid population, Northeast India. *Helicobacter* **21**, 523–535. 10.1111/hel.12308 (2016). 27006283 10.1111/hel.12308

[9] Sharma, N., Singh, A., Singh, N., Behera, D. & Sharma, S. Genetic polymorphisms in GSTM1, GSTT1 and GSTP1 genes and risk of lung cancer in a North Indian population. *Cancer Epidemiol.* **39**, 947–955. 10.1016/j.canep.2015.10.014 (2015). 26529288 10.1016/j.canep.2015.10.014

[10] Vilckova, M. et al. Variation in N-acetyltransferase 2 (NAT2), smoking and risk of prostate cancer in the Slovak population. *Med. Oncol.* **31**, 987. 10.1007/s12032-014-0987-3 (2014). 24816842 10.1007/s12032-014-0987-3

[11] Zhang, C. et al. Variants in CYP2J2 and CYP2C9 Contribute to Susceptibility of Lung Cancer. *Curr. Cancer Drug Targets*. 10.2174/1568009623666221114115012 (2022). 36380410 10.2174/1568009623666221114115012

[12] Bellamri, M., Xiao, S., Murugan, P., Weight, C. J. & Turesky, R. J. Metabolic activation of the cooked meat carcinogen 2-Amino-1-Methyl-6-Phenylimidazo[4,5-b]Pyridine in human prostate. *Toxicol. Sci.* **163**, 543–556. 10.1093/toxsci/kfy060 (2018). 29596660 10.1093/toxsci/kfy060 PMC5974788

[13] Jancova, P., Anzenbacher, P. & Anzenbacherova, E. Phase II drug metabolizing enzymes. *Biomed Pap Med Fac Univ Palacky Olomouc Czech Repub* **154**, 103–116. 10.5507/bp.2010.017 (2010). 20668491 10.5507/bp.2010.017

[14] Turesky, R. J. et al. Metabolism of the food-borne mutagen 2-amino-3,8-dimethylimidazo[4,5-f]quinoxaline in humans. *Chem. Res. Toxicol.* **11**, 217–225. 10.1021/tx9701891 (1998). 9544620 10.1021/tx9701891

[15] Piao, J. M. et al. Glutathione-s-transferase (GSTM1, GSTT1) and the risk of gastrointestinal cancer in a Korean population. *World J. Gastroenterol.* **15**, 5716–5721. 10.3748/wjg.15.5716 (2009). 19960570 10.3748/wjg.15.5716 PMC2789226

[16] Saadat, I. & Saadat, M. Glutathione s-transferase M1 and T1 null genotypes and the risk of gastric and colorectal cancers. *Cancer Lett.* **169**, 21–26. 10.1016/s0304-3835(01)00550-x (2001). 11410321 10.1016/s0304-3835(01)00550-x

[17] Clyne, M. & Rowland, M. The Role of Host Genetic Polymorphisms in Helicobacter pylori Mediated Disease Outcome. *Adv. Exp. Med. Biol.* **1149**, 151–172. 10.1007/5584_2019_364 (2019). 31016623 10.1007/5584_2019_364

[18] Priyadarshini, A., Chakraborti, A., Mandal, A. K. & Singh, S. K. Asp299Gly and Thr399Ile polymorphism of TLR-4 gene in patients with prostate cancer from North India. *Indian J. Urol.* **29**, 37–41. 10.4103/0970-1591.109982 (2013). 23671363 10.4103/0970-1591.109982 PMC3649598

[19] Cortes, J. E., Talpaz, M., Cabanillas, F., Seymour, J. F. & Kurzrock, R. Serum levels of interleukin-10 in patients with diffuse large cell lymphoma: Lack of correlation with prognosis. *Blood* **85**, 2516–2520 (1995). 7537119

[20] Gholamalizadeh, M. et al. The Role of Tumor Necrosis Factor-alpha (TNF-alpha) Polymorphisms in Gastric Cancer: a Meta-Analysis. *J. Gastrointest. Cancer*. **53**, 756–769. 10.1007/s12029-021-00688-w (2022). 34478034 10.1007/s12029-021-00688-w

[21] Sjoberg, J. et al. Interleukin-10 mRNA expression in B-cell chronic lymphocytic leukaemia inversely correlates with progression of disease. *Br. J. Haematol.* **92**, 393–400. 10.1046/j.1365-2141.1996.00358.x (1996). 8603006 10.1046/j.1365-2141.1996.00358.x

[22] Dressler, L. et al. Comparative assessment of genes driving cancer and somatic evolution in non-cancer tissues: An update of the Network of Cancer Genes (NCG) resource. *Genome Biol.* **23**, 35. 10.1186/s13059-022-02607-z (2022). 35078504 10.1186/s13059-022-02607-z PMC8790917

[23] Zhang, H. & Rosdahl, I. Expression of oncogenes, tumour suppressor, mismatch repair and apoptosis-related genes in primary and metastatic melanoma cells. *Int. J. Oncol.* **19**, 1149–1153. 10.3892/ijo.19.6.1149 (2001). 11713583 10.3892/ijo.19.6.1149

[24] Chen, S. Y. et al. Modification effects of GSTM1, GSTT1 and CYP2E1 polymorphisms on associations between raw salted food and incomplete intestinal metaplasia in a high-risk area of stomach cancer. *Int. J. Cancer* **108**, 606–612. 10.1002/ijc.11535 (2004). 14696128 10.1002/ijc.11535

[25] de Oliveira, J. G. & Silva, A. E. Polymorphisms of the TLR2 and TLR4 genes are associated with risk of gastric cancer in a Brazilian population. *World J. Gastroenterol.* **18**, 1235–1242. 10.3748/wjg.v18.i11.1235 (2012). 22468087 10.3748/wjg.v18.i11.1235 PMC3309913

[26] Shih, C. M. et al. The involvement of genetic polymorphism of IL-10 promoter in non-small cell lung cancer. *Lung Cancer* **50**, 291–297. 10.1016/j.lungcan.2005.07.007 (2005). 16122836 10.1016/j.lungcan.2005.07.007

[27] Singh, P. K. et al. Association of TNF-α (-238 and-308) promoter polymorphisms with susceptibility of oral squamous cell carcinoma in North Indian population. *Cancer Biomark.* **15**, 125–131. 10.3233/CBM-140444 (2015). 25519014 10.3233/CBM-140444 PMC12928520

[28] Jafarzadeh, A., Rezayati, M. T. & Nemati, M. Specific serum immunoglobulin G to H pylori and CagA in healthy children and adults (South-east of Iran). *World J. Gastroenterol.* **13**, 3117–3121. 10.3748/wjg.v13.i22.3117 (2007). 17589930 10.3748/wjg.v13.i22.3117 PMC4172621

[29] Shanker, N. et al. Cancer scenario in North-East India & need for an appropriate research agenda. *Indian J. Med. Res.* **154**, 27–35. 10.4103/ijmr.IJMR_347_20 (2021). 34782528 10.4103/ijmr.IJMR_347_20 PMC8715693

[30] Tan, D. S., Mok, T. S. & Rebbeck, T. R. Cancer genomics: Diversity and disparity across ethnicity and geography. *J. Clin. Oncol.* **34**, 91–101. 10.1200/JCO.2015.62.0096 (2016). 26578615 10.1200/JCO.2015.62.0096

[31] Servarayan Murugesan, C. et al. Gastric cancer in India: Epidemiology and standard of treatment. *Updates Surg.* **70**, 233–239. 10.1007/s13304-018-0527-3 (2018). 29611052 10.1007/s13304-018-0527-3

[32] Katoh, T. et al. Glutathione S-transferase M1 (GSTM1) and T1 (GSTT1) genetic polymorphism and susceptibility to gastric and colorectal adenocarcinoma. *Carcinogenesis* **17**, 1855–1859. 10.1093/carcin/17.9.1855 (1996). 8824506 10.1093/carcin/17.9.1855

[33] Hecht, S. S. & Hatsukami, D. K. Smokeless tobacco and cigarette smoking: Chemical mechanisms and cancer prevention. *Nat. Rev. Cancer.* **22**, 143–155. 10.1038/s41568-021-00423-4 (2022). 34980891 10.1038/s41568-021-00423-4 PMC9308447

[34] Sharan, R. N., Mehrotra, R., Choudhury, Y. & Asotra, K. Association of betel nut with carcinogenesis: Revisit with a clinical perspective. *PLoS One* **7**, e42759. 10.1371/journal.pone.0042759 (2012). 22912735 10.1371/journal.pone.0042759 PMC3418282

[35] Evans, S. C. & Lozano, G. The Li-Fraumeni syndrome: An inherited susceptibility to cancer. *Mol. Med. Today* **3**, 390–395. 10.1016/S1357-4310(97)01105-2 (1997). 9302689 10.1016/S1357-4310(97)01105-2

[36] Guha, T. & Malkin, D. Inherited TP53 mutations and the Li-Fraumeni Syndrome. *Cold Spring Harb. Perspect. Med.*10.1101/cshperspect.a026187 (2017). 28270529 10.1101/cshperspect.a026187 PMC5378014

[37] Varley, J. M. Germline TP53 mutations and Li-Fraumeni syndrome. *Hum. Mutat.* **21**, 313–320. 10.1002/humu.10185 (2003). 12619118 10.1002/humu.10185

[38] Groenewald, W., Lund, A. H. & Gay, D. M. The role of WNT pathway mutations in cancer development and an overview of therapeutic options. *Cells*10.3390/cells12070990 (2023). 37048063 10.3390/cells12070990 PMC10093220

[39] Sugimoto, S., Komatsu, H., Morohoshi, Y. & Kanai, T. Recognition of and recent issues in hereditary diffuse gastric cancer. *J. Gastroenterol.* **50**, 831–843. 10.1007/s00535-015-1093-9 (2015). 26049741 10.1007/s00535-015-1093-9

[40] De Luca, A., Maiello, M. R., D’Alessio, A., Pergameno, M. & Normanno, N. The RAS/RAF/MEK/ERK and the PI3K/AKT signalling pathways: Role in cancer pathogenesis and implications for therapeutic approaches. *Expert Opin. Ther. Targets* **16**(Suppl 2), S17-27. 10.1517/14728222.2011.639361 (2012). 22443084 10.1517/14728222.2011.639361

[41] Brait, M. et al. Comparative mutational landscape analysis of patient-derived tumour xenografts. *Br. J. Cancer* **116**, 515–523. 10.1038/bjc.2016.450 (2017). 28118322 10.1038/bjc.2016.450 PMC5318980

[42] Miwa, H. et al. Insufficient diagnostic accuracy of imported serological kits for *Helicobacter pylori* infection in Japanese population. *Diagn. Microbiol. Infect. Dis.* **36**, 95–99. 10.1016/s0732-8893(99)00143-1 (2000). 10705050 10.1016/s0732-8893(99)00143-1

[43] Williams, M. T. & Hord, N. G. The role of dietary factors in cancer prevention: Beyond fruits and vegetables. *Nutr. Clin. Pract.* **20**, 451–459. 10.1177/0115426505020004451 (2005). 16207684 10.1177/0115426505020004451

