# Supplementary material for: Genetic susceptibility and gene–environment interactions in gastric cancer among ethnic populations of Northeast India
Source: Sci Rep. 2026 May 6;16:20900. doi: 10.1038/s41598-026-50133-w (PMC13338060; doi:10.1038/s41598-026-50133-w)
Supplement: Supplementary file 1 — Supplementary Material 1 [file 41598_2026_50133_MOESM1_ESM.docx]

**Supplementary Table S5: Interaction of *GSTT1* polymorphism and betel nut chewing habit and risk of Gastric cancer**

| *GSTT1* and betel nut chewing habits | | Case | Control | Univariate logistic regression | | Multiple logistic regression | |
| --- | --- | --- | --- | --- | --- | --- | --- |
|  |  | n (%) | n (%) | OR (95% CI) | p-value | OR (95% CI) | p-value |
| Never chewer | Non-null | 56 (65.9) | 148 (64.3) | 1 |  | 1 |  |
|  | Null | 29 (34.1) | 82 (35.6) | 0.93 (0.55 – 1.58) | 0.800 | 1.21 (0.69 – 2.12) | 0.508 |
| Ever chewer | Non-null | 72 (68.5) | 71 (81.6) | 1 |  | 1 |  |
|  | Null | 33 (31.4) | 16 (18.4) | 2.03 (1.03 – 4.02) | 0.041* | 1.53 (0.73 – 3.19) | 0.256 |
| *Adjusted for age, sex and state in multiple logistic regression model*  **Significant P value* | | | | | | | |
