# Supplementary material for: Genetic susceptibility and gene–environment interactions in gastric cancer among ethnic populations of Northeast India
Source: Sci Rep. 2026 May 6;16:20900. doi: 10.1038/s41598-026-50133-w (PMC13338060; doi:10.1038/s41598-026-50133-w)
Supplement: Supplementary file 2 — Supplementary Material 2 [file 41598_2026_50133_MOESM2_ESM.docx]

**Supplementary Table S2. Lauren histological classification of gastric cancer cases (n = 161)**

| **Lauren type** | **n** | **%** |
| --- | --- | --- |
| Intestinal | 106 | 65.8 |
| Diffuse | 16 | 9.9 |
| Mixed | 21 | 13.0 |
| Unclassifiable/Other* | 18 | 11.2 |
| **Total** | **161** | **100.0** |

*Unclassifiable/Other includes cases where the pathology report did not explicitly state Lauren subtype.
