# Supplementary material for: Genetic susceptibility and gene–environment interactions in gastric cancer among ethnic populations of Northeast India
Source: Sci Rep. 2026 May 6;16:20900. doi: 10.1038/s41598-026-50133-w (PMC13338060; doi:10.1038/s41598-026-50133-w)
Supplement: Supplementary file 3 — Supplementary Material 3 [file 41598_2026_50133_MOESM3_ESM.docx]

**Supplementary Table S3: Risk of gastric cancer in tobacco chewers and betel nut chewers**

| Factors | Case  (n=190) | Control  (n=317) | Univariate logistic regression | | Multiple logistic regression | |
| --- | --- | --- | --- | --- | --- | --- |
|  | n (%) | n (%) | OR (95% CI) | p-value | OR (95% CI) | p-value |
| **Tobacco users** |  |  |  |  |  |  |
| Never | 141 (74.2) | 289 (91.2) | 1 |  | 1 |  |
| Ever | 49 (25.8) | 28 (8.8) | 3.59 (2.16 – 5.95) | **<0.001*** | 2.71 (1.52 – 4.83) | **0.001*** |
| **Betel nut chewing** |  |  |  |  |  |  |
| Never | 85 (44.7) | 230 (72.6) | 1 |  | 1 |  |
| Ever | 105 (55.3) | 87 (27.4) | 3.27 (2.24 – 4.76) | **<0.001*** | 2.24 (1.45 – 3.48) | **< 0.001*** |
| *Adjusted for age, sex and state in multiple logistic regression model*  **Significant P value* | | | | | | |
