# Supplementary material for: Genetic susceptibility and gene–environment interactions in gastric cancer among ethnic populations of Northeast India
Source: Sci Rep. 2026 May 6;16:20900. doi: 10.1038/s41598-026-50133-w (PMC13338060; doi:10.1038/s41598-026-50133-w)
Supplement: Supplementary file 4 — Supplementary Material 4 [file 41598_2026_50133_MOESM4_ESM.docx]

**Supplementary Table S6: Interaction of *GSTM1* polymorphism and betel nut chewing habit and risk of Gastric cancer**

| *GSTM1* and betel nut chewing habits | | Case | Control | Univariate logistic regression | | Adjusted logistic regression | |
| --- | --- | --- | --- | --- | --- | --- | --- |
|  |  | n (%) | n (%) | OR (95% CI) | p-value | OR (95% CI) | p-value |
| Never chewer | Non-null | 36 (43.9) | 128 (55.6) | 1 |  | 1 |  |
|  | Null | 46 (56.1) | 102 (44.3) | 1.71 (1.03 – 2.82) | 0.037* | 2.22 (1.29 – 3.82) | 0.004* |
| Ever chewer | Non-null | 51 (48.5) | 64 (73.6) | 1 |  | 1 |  |
|  | Null | 54 (51.4) | 23 (26.4) | 2.95 (1.60 – 5.43) | 0.001* | 2.57 (1.34 – 4.92) | 0.004* |
| *Adjusted for age, sex and state in the adjusted logistic regression model*  **Significant P value* | | | | | | | |
