# Supplementary material for: Genetic susceptibility and gene–environment interactions in gastric cancer among ethnic populations of Northeast India
Source: Sci Rep. 2026 May 6;16:20900. doi: 10.1038/s41598-026-50133-w (PMC13338060; doi:10.1038/s41598-026-50133-w)
Supplement: Supplementary file 5 — Supplementary Material 5 [file 41598_2026_50133_MOESM5_ESM.docx]

**Supplementary Table S7.** **Interaction of *CYP2E1* polymorphism and tobacco chewing habit and risk of Gastric cancer**

| *CYP2E1* and tobacco chewing habits | | Case | Control | Univariate logistic regression | | Multiple logistic regression | |
| --- | --- | --- | --- | --- | --- | --- | --- |
|  |  | n (%) | n (%) | OR (95% CI) | p-value | OR (95% CI) | p-value |
| Never chewer | C1/C1 | 129 (91.5) | 264 (94.0) | Reference |  | Reference |  |
|  | C1/C2 | 12 (8.5) | 17 (6.0) | 1.44 (0.67 – 3.11) | 0.348 | 2.03 (0.89 – 4.66) | 0.094 |
| Ever chewer | C1/C1 | 43 (87.7) | 27 (96.4) | Reference |  | Reference |  |
|  | C1/C2 | 6 (12.2) | 1 (3.6) | 3.77 (0.43 – 33.03) | 0.231 | 5.11 (0.46 – 56.81) | 0.184 |
| *Adjusted for age, sex and state in multiple logistic regression model* | | | | | | | |
