# Supplementary material for: Genetic susceptibility and gene–environment interactions in gastric cancer among ethnic populations of Northeast India
Source: Sci Rep. 2026 May 6;16:20900. doi: 10.1038/s41598-026-50133-w (PMC13338060; doi:10.1038/s41598-026-50133-w)
Supplement: Supplementary file 6 — Supplementary Material 6 [file 41598_2026_50133_MOESM6_ESM.docx]

| **Age_group** | **Number of patients** | **Total** | **Mean** | **Median** | **Min** | **Max** | **Mean age** |
| --- | --- | --- | --- | --- | --- | --- | --- |
|  |  |  |  |  |  |  |  |
| **Young (<40 years)** | **11** | **72** | **6.55** | **5** | **2** | **24** | **33.09** |
|  |  |  |  |  |  |  |  |
| **Middle (40 to 59 years)** | **33** | **129** | **3.91** | **4** | **0** | **9** | **49.76** |
|  |  |  |  |  |  |  |  |
| **Old (60 years & above)** | **36** | **169** | **4.69** | **4** | **1** | **15** | **69.56** |

**Supplementary Table S12. Age-group wise burden of mutations in gastric cancer patients (n=80)**
