# Supplementary material for: Genetic susceptibility and gene–environment interactions in gastric cancer among ethnic populations of Northeast India
Source: Sci Rep. 2026 May 6;16:20900. doi: 10.1038/s41598-026-50133-w (PMC13338060; doi:10.1038/s41598-026-50133-w)
Supplement: Supplementary file 7 — Supplementary Material 7 [file 41598_2026_50133_MOESM7_ESM.docx]

**Supplementary Table S10. Results of case control study based on logistic regression analysis depicting dietary factors and their association with gastric cancer**

| Factors | Case  (n=190) | Control  (n=317) | Univariate logistic regression | | Multiple logistic regression | |
| --- | --- | --- | --- | --- | --- | --- |
|  | n (%) | n (%) | OR (95% CI) | p-value | OR (95% CI) | p-value |
| **Frequency of consumption of vegetables and fruits per week** | | | | | | |
| **Pumpkin** |  |  |  |  |  |  |
| Occasionally | 136 (71.6) | 115 (36.3) | 1 |  | 1 |  |
| At least 2 times | 54 (28.4) | 195 (61.5) | 0.23 (0.16 – 0.35) | < 0.001* | 0.30 (0.17 – 0.50) | < 0.001* |
| **Cabbage** |  |  |  |  |  |  |
| Occasionally | 114 (60.0) | 110 (34.7) | 1 |  | 1 |  |
| At least 2 times | 74 (38.9) | 204 (64.4) | 0.35 (0.24 – 0.51) | < 0.001* | 0.58 (0.33 – 1.00) | 0.051* |
| **Cauliflower** |  |  |  |  |  |  |
| Occasionally | 100 (52.6) | 91 (28.7) | 1 |  | 1 |  |
| At least 2 times | 77 (40.5) | 186 (58.7) | 0.38 (0.26 – 0.56) | < 0.001* | 0.69 (0.38 – 1.27) | 0.238 |
| **Tomato** |  |  |  |  |  |  |
| Occasionally | 67 (35.3) | 59 (18.6) | 1 |  | 1 |  |
| At least 2 times | 122 (64.2) | 255 (80.4) | 0.42 (0.28 – 0.64) | < 0.001* | 0.59 (0.35 – 0.99) | 0.048* |
| **Fresh fruits** |  |  |  |  |  |  |
| Occasionally | 95 (50.0) | 92 (29.0) | 1 |  | 1 |  |
| At least 2 times | 95 (50.0) | 223 (70.3) | 0.41 (0.28 – 0.60) | < 0.001* | 0.44 (0.25 – 0.75) | 0.003* |
| *Adjusted for age, sex and state in multiple logistic regression model*  **Significant P value* | | | | | | |
