# Supplementary material for: Genetic susceptibility and gene–environment interactions in gastric cancer among ethnic populations of Northeast India
Source: Sci Rep. 2026 May 6;16:20900. doi: 10.1038/s41598-026-50133-w (PMC13338060; doi:10.1038/s41598-026-50133-w)
Supplement: Supplementary file 8 — Supplementary Material 8 [file 41598_2026_50133_MOESM8_ESM.docx]

**Supplementary Table S4.** **Interaction of *GSTT1* polymorphism and tobacco chewing habit and risk of Gastric cancer**

| *GSTT1* and tobacco chewing habits | | Case | Control | Univariate logistic regression | | Adjusted logistic regression | |
| --- | --- | --- | --- | --- | --- | --- | --- |
|  |  | n (%) | n (%) | OR (95% CI) | p-value | OR (95% CI) | p-value |
| Never chewer | Non-null | 98 (69.5) | 196 (67.8) | 1 |  | 1 |  |
|  | Null | 43 (30.5) | 93 (32.2) | 0.92 (0.59 – 1.43) | 0.725 | 1.12 (0.70 – 1.81) | 0.627 |
| Ever chewer | Non-null | 30 (61.2) | 23 (82.1) | 1 |  | 1 |  |
|  | Null | 19 (38.8) | 5 (17.9) | 2.91 (0.95 – 8.97) | 0.062 | 2.81 (0.82 – 9.58) | 0.099 |
| *Adjusted for age, sex and state in multiple logistic regression model*  **Significant P value* | | | | | | | |
