# Supplementary material for: Genetic susceptibility and gene–environment interactions in gastric cancer among ethnic populations of Northeast India
Source: Sci Rep. 2026 May 6;16:20900. doi: 10.1038/s41598-026-50133-w (PMC13338060; doi:10.1038/s41598-026-50133-w)
Supplement: Supplementary file 9 — Supplementary Material 9 [file 41598_2026_50133_MOESM9_ESM.docx]

**Supplementary Table S8. Interaction of *CYP2E1* polymorphism and betel-nut chewing habit and risk of Gastric cancer**

| *CYP2E1* and betel nut chewing habits | | Case | Control | Univariate logistic regression | | Multiple logistic regression | |
| --- | --- | --- | --- | --- | --- | --- | --- |
|  |  | n (%) | n (%) | OR (95% CI) | p-value | OR (95% CI) | p-value |
| Never chewer | C1/C1 | 76 (89.4) | 206 (92.8) | 1 |  | 1 |  |
|  | C1/C2 | 9 (10.6) | 16 (7.2) | 1.52 (0.65 – 3.60) | 0.335 | 2.43 (0.97 – 6.06) | 0.057 |
| Ever chewer | C1/C1 | 96 (94.1) | 85 (97.7) | 1 |  | 1 |  |
|  | C1/C2 | 9 (8.8) | 2 (2.3) | 3.98 (0.84 – 18.96) | 0.082 | 3.37 (0.60 – 19.07) | 0.168 |
| *Adjusted for age, sex and state in multiple logistic regression model* | | | | | | | |
