# Supplementary material for: Genetic susceptibility and gene–environment interactions in gastric cancer among ethnic populations of Northeast India
Source: Sci Rep. 2026 May 6;16:20900. doi: 10.1038/s41598-026-50133-w (PMC13338060; doi:10.1038/s41598-026-50133-w)
Supplement: Supplementary file 10 — Supplementary Material 10 [file 41598_2026_50133_MOESM10_ESM.docx]

**Supplementary Table S1. Demographic characteristics of the gastric cancer cases and healthy controls**

| Factors | Case (n=190) | Control (n=317) |
| --- | --- | --- |
|  | # (%) | # (%) |
| **Age** |  |  |
| Up to 40 | 28 (14.7) | 76 (24.0) |
| 41 to 60 | 88 (46.3) | 136 (42.9) |
| More than 61 | 74 (38.9) | 105 (33.1) |
| **Gender** |  |  |
| Female | 62 (32.6) | 113 (35.6) |
| Male | 128 (67.4) | 204 (64.4) |
| **Educational status** |  |  |
| Illiterate | 29 (15.3) | 35 (11.0) |
| Up to middle class | 111 (58.4) | 162 (51.1) |
| Up to Secondary | 30 (15.8) | 74 (23.3) |
| Graduate & above | 20 (10.5) | 45 (14.5) |
| **Occupational status** |  |  |
| Unemployed | 42 (22.1) | 109 (34.4) |
| Employed | 106 (55.8) | 138 (43.5) |
| Housewives | 42 (22.1) | 70 (22.1) |
|  |  |  |
| **Family History of cancer** |  |  |
| Any cancer | 21 (11.1) | 22 (6.9) |
| Gastric cancer | 8 (4.2) | 2 (0.6) |
|  |  |  |
| **Smoking status** | 76 (40.0) | 56 (17.7) |
