# Supplementary material for: Genetic susceptibility and gene–environment interactions in gastric cancer among ethnic populations of Northeast India
Source: Sci Rep. 2026 May 6;16:20900. doi: 10.1038/s41598-026-50133-w (PMC13338060; doi:10.1038/s41598-026-50133-w)
Supplement: Supplementary file 11 — Supplementary Material 11 [file 41598_2026_50133_MOESM11_ESM.docx]

**Supplementary Table S9. *TLR2* polymorphism and risk of gastric cancer**

| Genotypes | Case  (n=190) | Control  (n=317) | Univariate logistic regression | | Multiple logistic regression | |
| --- | --- | --- | --- | --- | --- | --- |
|  | n (%) | n (%) | OR (95% CI) | p-value | OR (95% CI) | p-value |
| *TLR2*Δ22 (-196-174 del) | | | | | | |
| I/I | 104 (54.7) | 153 (48.3) | 1 |  | 1 |  |
| I/D | 76 (40.0) | 138 (43.5) | 0.81 (0.56 – 1.18) | 0.271 | 0.96 (0.64 – 1.43) | 0.830 |
| D/D | 10 (5.3) | 26 (8.2) | 0.57 (0.26 – 1.22) | 0.148 | 0.82 (0.36 – 1.86) | 0.641 |
| *TLR4* (Asp299Gly) (rs 4986790) | | | | | | |
| A/A | 172 (90.5) | 269 (84.9) | 1 |  | 1 |  |
| A/G | 17 (8.9) | 44 (13.9) | 0.60 (0.33 – 1.09) | 0.095 | 0.44 (0.24 – 0.83) | 0.011 |
| G/G | 1 (0.5) | 4 (1.3) | 0.39 (0.04 – 3.53) | 0.403 | 0.29 (0.03 – 2.73) | 0.282 |
| *Adjusted for age, sex and state in multiple logistic regression model* | | | | | | |
